# The time-course of action control: measuring conditioned action tendencies and action suppression using transcranial magnetic stimulation

**DOI:** 10.1093/cercor/bhaf283

**Published:** 2025-10-15

**Authors:** Yvonne Y Chan, Dominic M D Tran, Justin A Harris, Evan J Livesey

**Affiliations:** School of Psychology, The University of Sydney, Sydney NSW, 2006, Australia; School of Psychology, The University of Sydney, Sydney NSW, 2006, Australia; School of Psychology, The University of Sydney, Sydney NSW, 2006, Australia; School of Psychology, The University of Sydney, Sydney NSW, 2006, Australia

**Keywords:** action control, associative learning, conditioned action tendency, conditioning, transcranial magnetic stimulation

## Abstract

Stimuli associated with a response can prime the motor system for action. While these action tendencies can be advantageous, reducing the demands of routine decisions and actions, they can be counter-productive when goals change. It is therefore important to understand how automatic motor preparation is brought under control. We used transcranial magnetic stimulation (TMS) to investigate the neurophysiological signatures of conditioned action tendencies in the motor system. Participants were trained to respond to target images appearing in a stream of other images. We then delivered TMS to the primary motor cortex to measure motor-evoked potentials (MEPs) as an index of cortico spinal excitability (CSE). Critically, participants were instructed to withhold the previously trained response to target cues. Despite this, the target cues increased CSE shortly before the timepoint at which a response would have typically been made (median RT). This was followed by distinct CSE suppression at later timepoints. TMS also triggered a motor response that may have otherwise been withheld in a time-sensitive manner. These results provide new evidence about the time-course of action tendencies triggered by conditioned cues and suggest that cue-elicited elevation of CSE is reined in when task goals change.

## Teaser

**Significance statement** Frequently repeated actions can become habitual and be elicited automatically by environmental cues they are associated with. These actions are executed more efficiently but become problematic when situational requirements change. Here, we map out the dynamic changes within the motor cortex as learned action tendencies are primed by environmental cues and then controlled. We show that a visual cue associated with a specific action provokes an initial increase in motor cortex excitability that can prime performance of the response even when it should be withheld. However, this action priming is quickly replaced by suppression of excitability in the same motor system reflecting control over the learned response. These temporal dynamics may be crucial for understanding how habitual tendencies are controlled.

## Introduction

When an action is performed repeatedly in the presence of the same cue, several well-documented behavioral changes occur. In the lab, this is observed as faster reaction times (RTs) and increased task accuracy (e.g. Tucker and Ellis 1998, 2004; Anderson et al. 2002). However, when task goals change and the response is no longer required, the same cue can provoke the response in error. In these situations, cognitive control is needed to override the previously learned tendencies (Botvinick et al. 2001; Prieto et al. 2023).

In healthy adults, overt behavioral errors can be rare and thus difficult to study in the laboratory under conditions where cognitive control is intact. However, in the absence of overt behavior, engagement of the motor system by stimuli associated with specific *actions* can be measured using transcranial magnetic stimulation (TMS). Delivering a single pulse of TMS to the hand region of primary motor cortex (M1) causes activation of corticospinal output neurons which can elicit contraction of the corresponding contralateral muscle. This muscle activity can be measured using electromyography (EMG). The size of the motor-evoked potential (MEP) elicited by the TMS provides an index of corticospinal excitability (CSE).

If TMS is delivered to M1 during tasks where the response requirement does not change, MEPs are increased in the presence of a cue associated with making a response (Poole et al. 2018; Tran et al. 2020a). This is also observed for cues associated with motor action in everyday life—MEPs are increased when participants see objects associated with specific manual responses (McNair et al. 2017; Cardellicchio et al. 2011; see also Freeman et al. 2016; Franca et al. 2012; Makris et al. 2011; Grafton et al. 1997).

Conditioned action tendencies may be counterproductive in situations where the task has changed to bring prior learning and current task goals into conflict. For example, Tran et al. (2019) used a task in which participants repeatedly responded by pressing a key to target letters appearing in a continuous sequence of other letters. After this training, the letter associated with the dominant hand response elevated CSE in the dominant hemisphere 300 ms after cue onset, even though participants were only counting the target letters and were no longer making key-press responses. When participants were required to make responses to *other* letters, but not the original targets, CSE was suppressed during the previously trained target letters. Tran et al. (2021) subsequently showed a similar suppression effect in a go/no-go task: a stimulus that had consistently required a response during training, but then became a no-go stimulus in the transfer task, suppressed CSE 300 ms after cue onset during the transfer task. This suppression effect was reduced when participants were placed under working memory load, implicating resource-dependent cognitive processes. The authors speculated that MEP suppression reflected control over motor engagement by target cues, preventing inappropriate actions (Tran et al. 2019, 2021).

While cues associated with responding can provoke both elevation and suppression of CSE depending on task goals, much less is known about the time course of these processes when goals change. For example, does a change in task goals produce an immediate shift from excitation to suppression or does that shift unfold within the course of a single trial, revealing both CSE excitation and suppression? This gap was addressed in the current study. Based on past research showing increased excitability of the agonist M1 representation in the 100 ms preceding EMG onset (Chen et al. 1998; Chen and Hallett 1999; Leocani et al. 2000; MacKinnon and Rothwell 2000; Hashimoto et al. 2004; McMillan et al. 2004), we predicted that CSE would be elevated by a previously trained response cue, reflective of a conditioned action tendency. Our primary objective was to see how long this elevation would persist and whether it might ultimately give way to CSE suppression once cognitive control processes are engaged to prevent the no-longer-required response (Tran et al. 2019, 2021).

## Materials and methods

Across three experiments, participants first completed multiple sessions of a continuous performance task in which they responded quickly to two target images as well as images of animals. In the test phase, participants were instructed to continue to respond to animals but not to the target images. TMS was delivered to M1 on target trials at key timepoints relative to the participant’s median RT during the previous training block.

In Experiment 1, we compared CSE at timepoints 100 ms before and after the participant’s median RT. In Experiment 2, we further investigated the time course of CSE elevation by testing time-points 150 ms and 50 ms prior to median RT. Experiment 3 provided a more complete within-experiment investigation of the time course of conditioned action tendencies and their suppression.

### Participants

The protocol used for all three experiments was approved by the Human Research Ethics Committee of The University of Sydney (Project No. 2016/920). All participants completed a TMS safety screening questionnaire and provided informed consent before commencing the experiment. Participants were excluded if they did not complete online training as instructed or for failing to respond to at least 80% of target or animal trials requiring a response, averaged across all four training sessions. We use this criterion to ensure a consistent level of engagement during training, in what is a relatively easy task but one that is completed in an unmonitored environment. We chose 80% accuracy based on piloting and previous research using go/no-go tasks run in our laboratory (e.g. Lee Cheong Lem et al. 2018). Additionally, participants were excluded from analysis if their MEPs were too small to be included (i.e. < 50 μV) on more than 50% of trials in any condition, and one participant in each of experiment was excluded because their mean MEP was more than 3 standard deviations from the mean of the remaining participants. We aimed to collect sufficient data to have approximately 40 data sets for each experiment, based on similar work in our lab and piloting. A sample of 40 participants provides 80% power to detect a medium-to-large effect size of d = 0.45.

#### Experiment 1

Forty-five undergraduate psychology students from the University of Sydney psychology undergraduate pool completed the online training for Experiment 1 and participated in exchange for course credit. Two participants were excluded for failing to meet the training criterion described above. In addition, another participant withdrew from the study due to discomfort with the TMS, and a fourth was excluded as an outlier because their MEPs were more than three standard deviations above the mean of the remaining participants. Analyses were conducted on the final sample of 41 participants (29 female; mean age = 19.0 years, SD = 1.68; 39 right-handed).

#### Experiment 2

Forty-seven participants completed all online training sessions for Experiment 2: 40 participants from the University of Sydney took part in the experiment in exchange for course credit, and an additional 7 participants were financially remunerated for their time. Of the 47 participants, 4 did not meet the training criterion described above. One participant was excluded for insufficient MEPs, and another was excluded for outlier MEP amplitudes. Due to technical issues, an additional 2 participants did not complete the experiment. All analyses were conducted on the remaining sample of 39 participants (28 female; mean age = 20.4 years, SD = 3.25; 36 right-handed).

#### Experiment 3

Forty-eight undergraduate University of Sydney psychology students completed all online training and participated in Experiment 3 in exchange for course credit. Three participants failed to show for the in-lab session. One participant was ineligible for TMS upon arrival to the in-lab session and another two withdrew from the study due to discomfort with the TMS. An additional three participants did not continue with the in-lab session due to high RMTs. From the remaining sample, an additional seven participants were excluded for having insufficient MEPs. Analyses were conducted on the final sample of 32 participants (25 female; mean age = 19.7 years, SD = 1.42; 29 right-handed).

### Apparatus and stimuli

The online training task was programmed and administered using Inquisit™ (v 6.4) and was completed using participants’ personal computers. The lab session was administered on a PC connected to a 24-inch Asus monitor (1920 x 1080 resolution, 60 Hz refresh rate) at a viewing distance of approximately 57 cm. PsychoPy (v2022.2.4) was used to control stimulus presentation and TMS delivery and collect behavioral response data.

The visual stimuli used in both the online training and in-lab tests consisted of 164 images (124 filler objects and 40 animals) selected from a colourised pictorial set created by Rossion and Pourtois (2004). Four images (banana, button, car, tree) were selected as target and control images on the basis of being semantically and visually distinct. Their allocation as target or control cues was counterbalanced across participants based in a Latin-square design. Stimuli were displayed on a 281 × 197-pixel white background (6.9° x 5.0° of visual angle, during the lab session) in the centre of a gray screen. Three auditory stimuli were used as feedback, as described below.

#### TMS/EMG

In all three experiments, TMS was administered using a Magstim™ (Whitland, UK) stimulator with a 70-mm figure-eight coil. The three experiments were run in different rooms with sightly different set-ups: a BiStim^2^ machine configured in simultaneous mode was used in Experiment 1; a Magstim 200^2^ machine in Experiment 2; and a BiStim^2^ configured in independent firing mode in Experiment 3. Importantly, resting motor threshold was determined by the same criteria (see below). Surface EMG traces were recorded from the first dorsal interosseous (FDI) muscle of the participant’s dominant hand. The skin was cleaned with an abrasive sponge and wiped with 70%v/v isopropyl alcohol before a pair of Ag/AgCl electrodes were placed over the FDI muscle and a ground electrode placed over the ulnar styloid process of the wrist. EMG activity was recorded using a PowerLab 26 T DAQ (ADInstruments™, Bella Vista, Australia) and digitally converted (sampling rate: 4 kHz, bandpass filter: 0.5 Hz to 2 kHz, mains filter: 50 Hz, and anti-aliasing) using LabChart™ software (Version 8, ADInstruments) for offline analysis.

TMS pulses were delivered to M1 contralateral to the dominant hand. Participants wore a fitted cap marked with the 10–20 EEG locations. The coil was positioned tangentially to the scalp with the handle orientated approximately 45° from the midline. The location of the motor hotspot was determined starting from a spot 5 cm lateral and 1 cm anterior to Cz and moving the coil around until the maximal MEP was elicited in the FDI muscle of the participant’s dominant hand. Once the hotspot was located, participants stabilized their head in an adjustable forehead and chin rest to minimize head movements, and the coil was locked in position using an adjustable variable friction arm (Manfrotto™, Cassola, Italy).

Resting motor threshold (rMT) was defined as the minimum stimulator intensity required to elicit an MEP with a peak-to-peak amplitude of > 50 μV in 5 out of 10 consecutive trials (Rossini et al. 2015). The mean rMT was 40.341 for Experiment 1 (BiStim^2^ in simultaneous mode), 43.692 for Experiment 2 (Magstim 200^2^), and 48.438 for Experiment 3 (BiStim^2^ in independent firing mode). Stimulation intensity was set to 120% of participant’s RMT for the duration of the experiment.

### Procedure

**Fig. 1:**
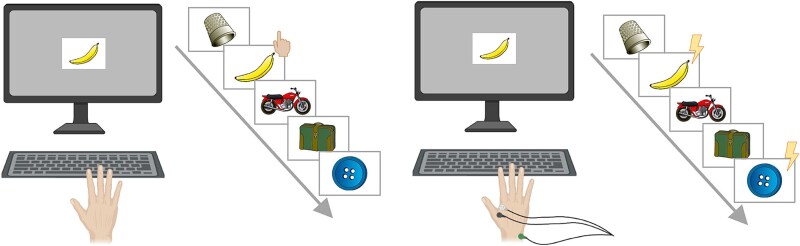
Illustration of the experimental task. The left figure shows the online training task in which participants saw a sequential stream of images and had to responding by pressing a key as soon as they saw a target image (e.g. banana) or an animal. The right figure illustrates the task during the test session when participants received TMS pulses that coincided with the target image and control image (e.g. button) while EMG recordings were made from their dominant hand.

The experiment consisted of two components: an online training phase involving four 10-min training sessions over four separate days, followed by an in-lab session scheduled one week after participants received the link to the initial training session. An illustration of the online task is shown in the left panel of Fig. 1.

#### Online training

Participants completed an online training phase consisting of four 10-min training sessions over four separate days, prior to attending the in-lab session. To ensure online training sessions were adequately spaced, links to the subsequent session were emailed only after completion of the previous session. During each of four online training sessions, participants viewed a stream of images (each displayed for up to 1000 ms) and were instructed to respond by pressing the spacebar with the index finger of their dominant hand as soon as they saw either of two target images (T1 and T2) as well as any images of animals. With the exception of the initial session, which began with a 1-min practice block, each session consisted of 24 presentations of each of T1 and T2 and each of two control images (C1 and C2) that did not require a response. The sessions also contained 24 presentations of animal images and 480 presentations of filler images, randomized and intermixed within the image stream. Control images were included to provide a measure of baseline responding and motor excitability during the TMS session.

To encourage speeded responses, keypresses made < 500 ms after stimulus onset were rewarded with 100 points, whereas keypresses made > 500 ms were rewarded with 10 points. Keypress responses to animals were also rewarded based on RT: 50 points for < 300 ms, 40 points for 300-399 ms, 30 points for 400-499 ms, 20 points for 500-599 ms, 10 points for 600-799 ms, 5 points for 800-899 ms and 1 point for any responses made ≥900 ms). Points feedback was displayed as text (e.g. “+50”) and accompanied by a chime (for responses < 500 ms) or ping (for responses ≥500 ms) sound. A penalty of 10 points was applied for failing to respond to a stimulus requiring a response (“Too slow! -10 points”) and accompanied by an error sound. For stimuli that did not require a response (i.e. neither target nor animal), the image was presented for 1000 ms and immediately followed by the next stimulus (no inter-stimulus interval). However, if the participant responded (in error) the image disappeared immediately and was replaced with an error message saying “Oops, No response required. Time out.” lasting 2000 ms. For stimuli requiring a response (animal or target), the image would appear for a maximum of 1000 ms or until the participant responded, at which point the image would disappear and was immediately replaced by feedback (as described above) for 500 ms.

#### In-lab session

For the final session in the TMS laboratory, participants first completed a refresher block of the training task. This was identical to the training sessions except for its shorter duration. The refresher block consisted of 16 each of T1, T2, C1, C2 and animals randomized and intermixed amongst 320 presentations of filler images. As in the training sessions, participants continued to respond to any targets and animals by pressing the spacebar with the index finger of their dominant hand. The refresher block allowed for estimation of median RT to determine timepoints for TMS delivery at test.

EMG and TMS apparatus were then set up in preparation for the test phase. During the test phase, participants viewed a similar stream of images as in training. Before beginning, they were instructed to withhold their responses to previously trained target images but continue to respond to images of animals. While feedback was maintained across animal trials as in training, incorrect responses to key stimuli of interest (T1, T2, C1, C2) were no longer followed by visual and auditory feedback. Stimuli of interest were separated from each other by at least 5 other images to ensure adequate time for the TMS machine to recharge, and separated from animal images by at least 1 filler to prevent interference in EMG activity by performance of a response.

For Experiments 1 and 2, each of the four test blocks consisted of 12 presentations each of T1, T2, C1, and C2, as well as 40 presentations of animal images, to match for rate of responding between training and test. Of the 12 presentations or each T and C image in each block, eight coincided with a TMS pulse delivered at one of two timepoints (4 pulses at each). The timepoints were 100 ms before or 100 ms after median RT (Experiment 1), and 150 ms or 50 ms before median RT (Experiment 2). The remaining four presentations of each stimulus of interest did not coincide with TMS delivery, and so allowed us to assess response likelihood in the absence of TMS.

Experiment 3 consisted of five test blocks each containing 10 presentations each of T1, T2, C1, and C2. These 10 presentations coincided with a TMS pulse delivered at one of 10 timepoints: −200, −150, −100, −50, 0, +50, +100, +200, +300, +400 ms from median RT. Additionally, there were 32 presentations of animal images and 200 filler images per block.

After completion of the TMS session, participants completed a stop signal task (SST) based on that used by Tran et al. (2024) and following analysis steps recommended by Verbruggen et al. (2019). This SST was added to address an exploratory research question—whether elevation and suppression of CSE were related to stopping efficiency—and was included because we have consistently found that other TMS-derived measures of inhibition correlate with stop-signal reaction time (e.g. Chowdhury et al. 2018, 2021; Tran et al. 2020b). However, we found no consistent relationships in this study, and therefore the methods and results from the SST are reported in supplemental materials only (see Figure S1).

### Data analysis

Since the two targets (T1 and T2) are functionally identical and response accuracy across target identities are equivalent across all stages of the experiment, all analyses are collapsed across targets. Likewise, we collapsed data across the two equivalent control images (C1 and C2).

The MEP data were pre-processed using custom Python software (https://github.com/nicolasmcnair/MEPAnalysis). MEP amplitudes were calculated as the difference between the highest and lowest points of activity within the MEP waveform. All trials were visually inspected to exclude trials with background activity in the 100 ms pre-TMS period (EMG activity > 50 μV). Any MEPs < 50 μV were also excluded from analysis. Participants were excluded for having MEPs ≥50 μV on fewer than 50% of trials for any stimulus at each TMS timepoint (see Table S2 in Supplemental Materials for number of trials included versus excluded per condition).

Behavioral responding was analyzed using a two factor (stimulus type × TMS time) ANOVA, with further pairwise comparisons between trials with TMS delivery and trials with no TMS, to identify the effect of TMS delivery on commission error rate (responding when no response was required). We used an alpha level of 0.05 for all statistical tests except for cases where a Bonferroni correction has been applied to correct for multiple comparisons.

Raw MEPs are reported for transparency, but all analyses were conducted on normalized data. Mean MEP amplitudes for the trained response stimuli (T1, T2) were normalized against mean MEP amplitudes to the non-trained controls (C1, C2) to provide an index of relative excitability and account for individual differences in absolute MEPs (Tran et al. 2019; Tran et al. 2021): MEP amplitudes towards target stimuli were divided by MEP amplitudes towards control stimuli and log transformed (log MEP = ln(MEPtarget/MEPcontrol). MEPs were then analyzed with one-sample t-tests to identify the effects of prior learning on relative CSE.

We also examined pre-TMS activity for each of the three experiments, to ascertain whether differences in muscle activity immediately before the TMS pulse may contribute to differences in MEP amplitudes. To measure pre-TMS activity, we took the root mean squared difference (RMSD) in the EMG record across the period spanning 100 ms to 5 ms before the TMS pulse. We note the outcome of these analyses briefly for each experiment (see Supplemental Materials for full statistical analyses).

## Results

### Online training

Response accuracy during training was high across all experiments. Mean accuracy (% correct) and RTs for each experiment are shown in Table 1.

**Table 1:** Behavioral data from online training in each experiment. Each row shows accuracy (% correct) and RTs to targets (T1 and T2) and animal images, as well as commission errors (% of filler images that elicited a key-press response).

	Target responding (final training session)		Animal responding (final training session)		Commission errors (across sessions)
	Accuracy (%)	RT (ms)	Accuracy (%)	RT (ms)	Error rate (%)
Experiment 1	97.97 (4.29)	470.9 (56.5)	97.33 (5.61)	512.1 (62.1)	0.69 (0.08)
Experiment 2	97.61 (5.38)	493.0 (69.5)	95.10 (7.91)	543.5 (87.0)	0.75 (0.09)
Experiment 3	97.99 (5.65)	469.0 (56.0)	96.73 (7.6)	524.2 (63.2)	0.61 (0.25)

### In-lab refresher block

Response accuracy remained high during the initial in-lab refresher block. The data for accuracy and RTs are presented in Table 2. Note that RTs were substantially faster in the refresher session than in online training. This may be due to several factors including software differences, computing hardware differences, motivation, level of distraction and other situational factors. The one-on-one testing environment is likely to increase participants’ motivation to perform (e.g. see Tran et al. 2024), and the timing precision from this session would also be higher. Note that the median RT value used for determining TMS timing was estimated from the laboratory refresher block.

**Table 2:** Behavioral data from the in-lab refresher block in each experiment. Each row shows accuracy (% correct) and RTs to targets (T1 and T2) and animal images, as well as commission errors (% of filler images that elicited a key-press response).

	Target responding (refresher)		Animal responding (refresher)		Commission errors (refresher)
	Accuracy (%)	RT (ms)	Accuracy (%)	RT (ms)	Error rate (%)
Experiment 1	100.00 (0.00)	416.6 (26.0)	99.70 (1.36)	459.4 (46.1)	0.03 (0.03)
Experiment 2	99.75 (1.52)	423.1 (31.3)	98.68 (2.96)	464.6 (45.6)	0.04 (0.04)
Experiment 3	100.00 (0.00)	420.5 (21.9)	99.41 (1.85)	467.1 (35.3)	0.28 (0.34)

### Test phase

#### Experiment 1

**Fig. 2:**
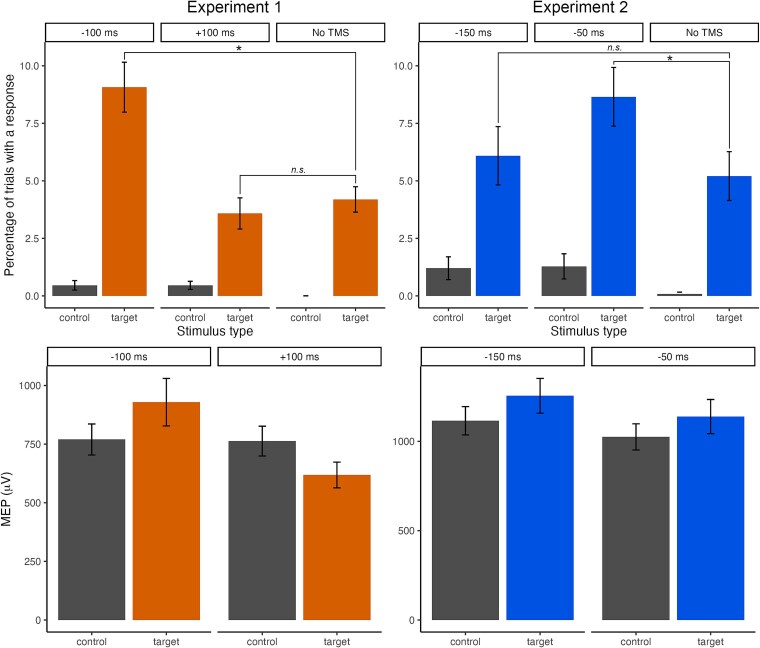
Response rates (proportion of trials with a response; top) and raw MEP amplitudes (bottom) during presentations of target or control images, as a function of TMS timing, in experiments 1 and 2. *Note*. Error bars show standard error of the mean.

During the test phase, participants were instructed to withhold keypress responses to T1 and T2 but to continue to respond to images of animals. Participants continued to respond to animals with high accuracy (mean = 99.47%, sd = 0.82; mean RT = 489.7 ms, sd =39.2). As shown in Fig. 2, a small proportion of response errors were committed on target and control presentations. A repeated-measures ANOVA conducted on the response rate to targets and controls revealed a significant main effect of stimulus type, *F*(1,40) = 97.383, *P* < 0.001, *η_p_^2^* = 0.709, such that participants made significantly more responses towards previously trained target images compared to controls which were never associated with responding. Additionally, a significant main effect of TMS time, *F*(2,80) = 18.093, *P* < 0.001, *η_p_^2^* = 0.311, and a significant interaction of stimulus type and TMS time, *F*(2,80) = 14.296, *P* < 0.001, *η_p_^2^* = 0.263, indicate that the increase in commission error rates was dependent on the timing of TMS delivery. To follow up this interaction, we used two paired t-tests, with a Bonferroni-adjusted α set to 0.025, to compare response rate at each TMS time (−100 or + 100 ms) against the response rate on no-TMS trials. A TMS pulse at −100 ms from median RT was associated with a significantly greater commission error rate compared to trials without TMS delivery, *t*(40) = 4.341, *P* < 0.001, *d* = 0.678. By contrast, responding on trials with TMS delivery at +100 ms from median RT was not different to trials without TMS, *t*(40) = −0.82186, *P* = 0.416, *d* = −.128. This suggests that the delivery of a TMS pulse shortly prior to median RT triggers the release of a response that would otherwise have been successfully withheld in the absence of TMS delivery. The mean response rates to T and C images as a function of TMS timing are shown in Fig. 2.

**Fig. 3:**
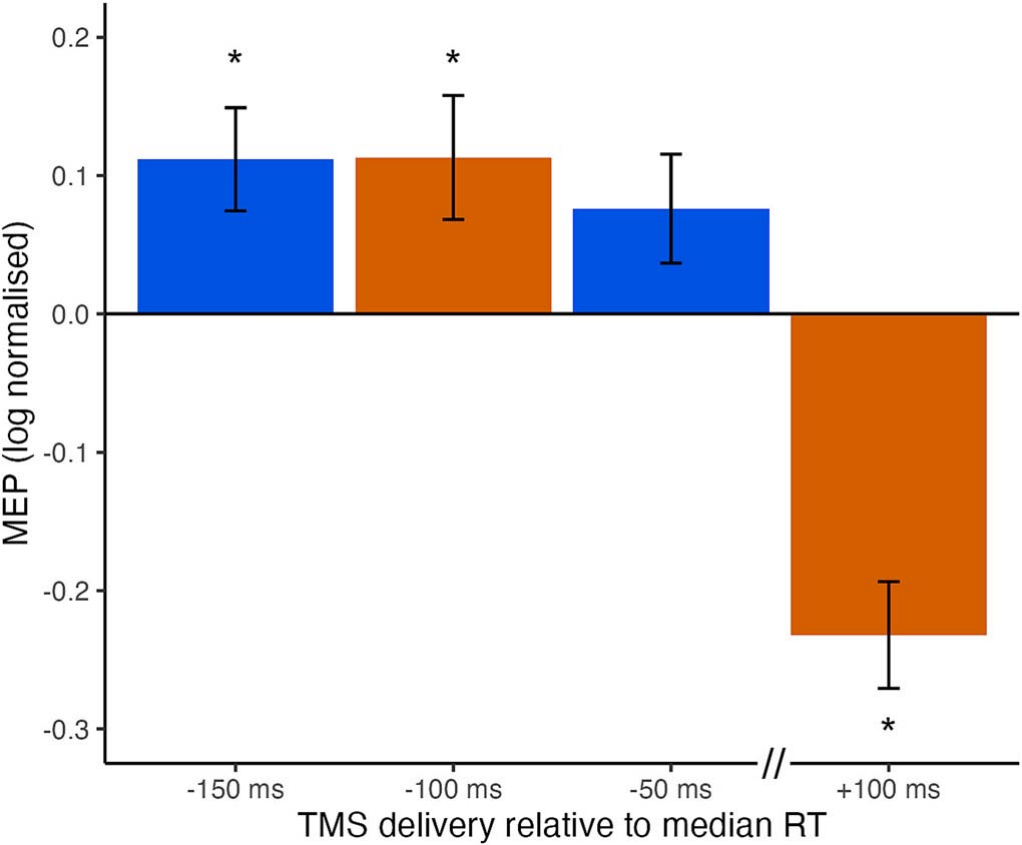
Log normalized MEPs during presentations of target images as a function of TMS timing across experiment 1 (−100 and + 100 ms) and experiment 2 (−150 and − 50 ms). *Note*. Error bars represent standard error of the mean.

Neurophysiological data from the test phase revealed opposing influences on CSE when TMS delivery occurred shortly before or after median RT. Consistent with the behavioral evidence, we observed neurophysiological evidence of conditioned action tendencies at the early stimulation timepoint (−100 ms) despite instruction to withhold responding. As shown in Fig. 2, MEPs were larger during target images than control images when TMS was delivered 100 ms before median RT, whereas MEPs were smaller during target than control images when TMS was delivered 100 ms after median RT. These effects were confirmed by statistical analyses on the log normalized MEPs (Fig. 3). Log normalized MEPs were significantly elevated when TMS was delivered at −100 ms from median RT, *t*(40) = 2.522, *P* = 0.016, *d* = 0.394, but were suppressed below baseline when TMS was delivered +100 ms after median RT, *t*(40) = −6.012, *P* < 0.001, *d* = −.939. That is, whilst conditioned motor priming was initially apparent after presentation of a target (shortly prior to median RT), these conditioned action tendencies come to be suppressed below baseline when participants withhold the trained response. Analyses of pre-TMS RMSD indicated no differences according to TMS timing or stimulus type (see Supplemental Materials).

#### Experiment 2

In the test phase, participants continued to respond to animals with high accuracy (mean = 98.46%, sd = 2.92; mean RT = 504.8 ms, sd = 49.5). As in Experiment 1, significant main effects of both stimulus type, *F*(1,38) = 41.500, *P* < 0.001, *η_p_^2^* = 0.522, and TMS time, *F*(2,76) = 5.415, *P* = 0.006, *η_p_^2^* = 0.125, indicate that participants made significantly more commission errors towards target stimuli than control images, and that commission error rate was dependent on timing of TMS delivery, although there was no interaction between these factors, *F*(2,76) = 2.252), *P* = 0.112, *η_p_^2^* = 0.056; see Fig. 2. Further analyses on the effect of TMS delivery time revealed that, when compared against an α level of 0.025, participants made significantly more commission errors towards targets on trials with TMS delivery 50 ms prior to median RT compared to trials without a TMS pulse, *t*(38) = 2.538, *P* = 0.015, *d* = 0.406, but a comparable level of commission errors on target trials where TMS was delivered 150 ms prior to median RT and target trials without a TMS pulse, *t*(38) = 0.681, *P* = 0.500, *d* = 0.109. This provides further evidence that the delivery of a TMS pulse shortly prior to median RT can interact with prior learning and trigger responding that would not otherwise have occurred.

Neurophysiological data from the test phase confirm evidence for motor priming by response-associated targets. MEPs were larger during presentation of a target image than a control image (Fig. 2). Consistent with increased commission errors towards trained targets, log normalized MEPs were significantly greater than 0 across TMS conditions, *t*(38) = 3.048, *P* = 0.004, *d* = 0.488. However, the effect of TMS timing on CSE did not parallel that of commission error rates. That is, there was a significant elevation in MEPs towards targets compared to controls when TMS was delivered −150 ms from median RT, *t*(38) = 2.994, *P* = 0.005, *d* = 0.479, but log normalized MEPs were not significantly elevated when TMS was delivered −50 ms from median RT, *t*(38) = 1.929, *P* = 0.061, *d* = 0.309. Nonetheless, the results of Experiment 2 confirm that TMS delivered during a former response target and shortly before the time when a response would have been made can produce elevated MEPs and trigger occasional responses, as observed in Experiment 1. Analyses of pre-TMS RMSD indicated that muscle activity was slightly elevated for target trials compared to control trials, but only for the later −50 ms timepoint. At the earlier −150 ms timepoint, where we saw strong evidence of an effect of stimulus type, there was no difference in pre-TMS activity comparing target and control trials (see Supplemental Materials).

#### Experiment 3

**Fig. 4:**
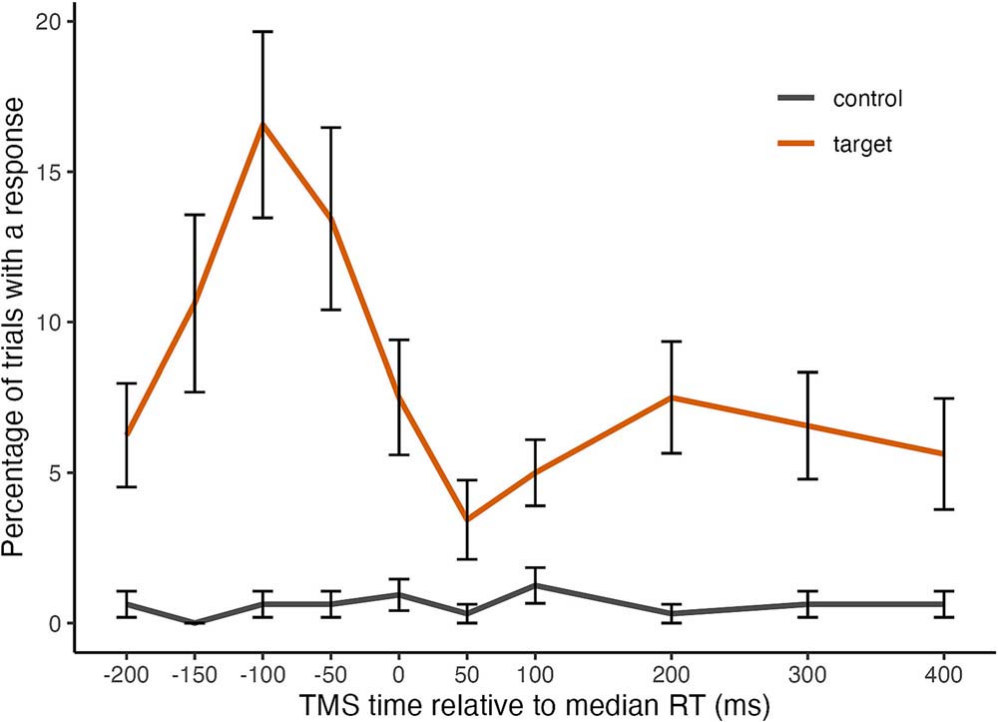
Response rate (proportion of trials with a response) during presentation of a target image or control image as a function of the timing of the TMS pulse in experiment 3. *Note*. Error bars represent standard error of the mean.

In the test phase, participants continued to respond to animals with high accuracy (mean = 98.77%, sd = 1.51; mean RT = 512.1 ms, sd = 41.7). As with Experiments 1 and 2, we conducted a repeated measures ANOVA with factors of stimulus type and TMS timing to test how the rate of commission errors is affected by stimulus presentation (target vs control image) and timing of the TMS pulse (see Fig. 4). This analysis revealed a significant main effect of stimulus type on responding, *F*(1,31) = 33.016, *P* < 0.001, *η_p_^2^* = 0.516, again indicating that participants made more commission error responses during trained targets than control images. There was also a significant main effect of TMS time, *F*(9,279) = 5.581, *P* < 0.001, *η_p_^2^* = 0.153, and a significant stimulus type by TMS timing interaction, *F*(9,279) = 5.511, *P* < 0.001, *η_p_^2^* = 0.151, indicating that the effect of TMS delivery on the commission error rate was again dependent on stimulus type. These results confirm that, rather than simply producing a response directly, TMS delivery interacts with prior learning to trigger responding.

**Fig. 5:**
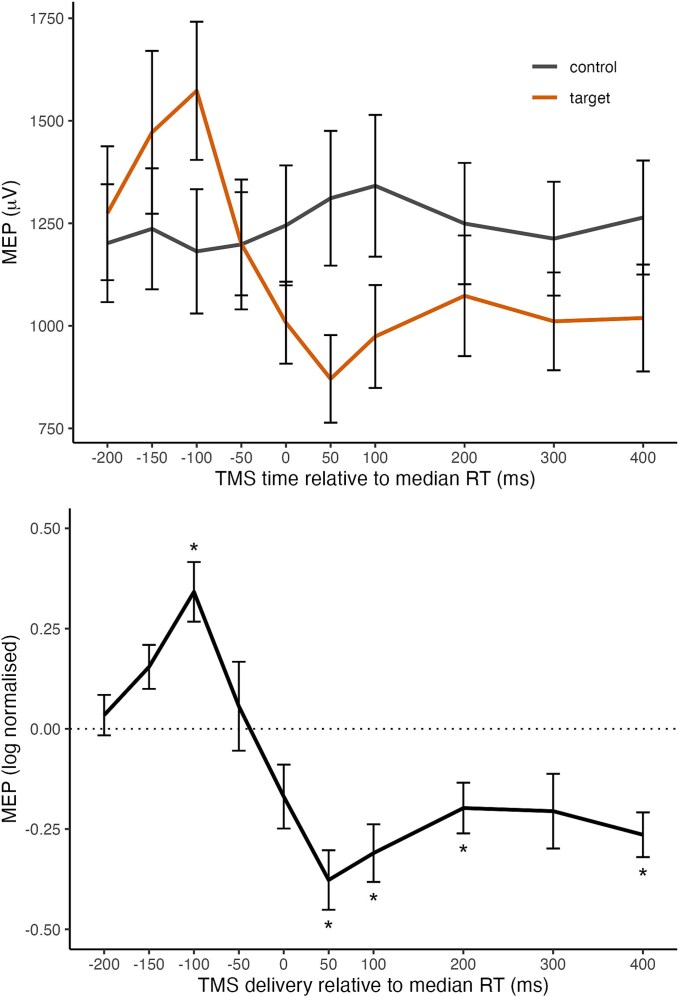
Raw MEP amplitudes during presentation of a target image or control image (left) and log normalized MEPs (right) during presentation of a target image as a function of the timing of the TMS pulse in experiment 3. *Note.* Error bars represent standard error of the mean. Asterisks represent significant comparisons against a Bonferroni adjusted alpha value of 0.005. See supplemental materials for detailed statistics.

Raw MEPs and log normalized MEPs are shown in the top and bottom panels, respectively, of Fig. 5, as a function of TMS timing in Experiment 3. These show that corticospinal excitability reflected the pattern observed in the behavioral measure and additionally provided evidence of a prolonged suppression effect after the time point at which a response would have otherwise been made. A univariate ANOVA on log normalized MEPs showed a significant effect of TMS timing, *F*(9,279) = 11.072, *P* < 0.001, *η_p_^2^* = 0.263. This was followed by a series of 10 single-sample t-tests against a log-normalized value of 0 for each TMS timepoint, with a Bonferroni correction to α at 0.005 (see Table S1 in Supplemental materials for full reporting). This showed that log normalized MEPs were only significantly above baseline (zero) when TMS was delivered 100 ms prior to median RT, *t*(31) = 4.594, *P* < 0.001, *d* = 0.812. This increase in CSE was quickly counteracted, with log normalized MEPs falling significantly below zero when TMS was delivered 50 ms after median RT, *t*(31) = −5.079, *P* < 0.001, *d* = −.898. This suppression effect persisted up to 400 ms after median RT, *t*(31) = −4.739, *P* < 0.001, *d* = −.838. Analyses of pre-TMS RMSD indicated no differences according to TMS timing or stimulus type (see Supplemental Materials). Together, these results provide evidence for dynamic changes in conditioned action tendencies reflected in (1) an increase in CSE in the period shortly before the time point at which a response would have otherwise been made, which (2) decreases to baseline around median RT, before (3) evolving into suppression of CSE that persists beyond the time when the response would have been made.

Our primary analysis reported above included trials with a commission error (see Supplemental Materials for a justification of this decision). Although these error trials are relatively infrequent, they may artificially inflate the amplitudes of MEPs under specific conditions, for instance if the participant is about to execute a planned and deliberate response (e.g. see Poole et al. 2018). This could result in artificially elevated CSE in the conditions with higher response rates, which in our experiments was typically on target stimulus presentations with early TMS at or around the −100 ms time point. To confirm that the CSE elevation for target trials at early time points was not due to these commission errors, we performed an aggregate analysis in which we took all data from the −150 and − 100 ms timepoint trials across all three experiments (*n* = 112), removed all trials that resulted in a commission error, and calculated log normalized MEP to gauge the degree to which MEPs were elevated for target trials relative to control trials. This analysis yielded significant elevation from baseline (i.e. 0), *t*(111) = 3.599, *P* < 0.001, *d* = 0.340, indicating overall strong evidence of CSE facilitation. These log normalized scores also correlated significantly with response rate to the target, *r* = 0.19, *P* = 0.047, *df* = 110. Note that this is most likely an *underestimate* of the true extent of CSE facilitation at these early timepoints (see Supplemental Materials).

## Discussion

The 3 experiments in this study addressed how action tendencies are brought under control when task goals change. Specifically, we investigated how the motor system responds to a stimulus that has been repeatedly associated with a specific action, but under conditions where that action is no longer appropriate. Using TMS to reveal action tendencies that would normally not be evident from responding alone, we investigated the time course of action control relative to the individual’s history of prior responding (relative to their median RT). Over three experiments, we found a clear pattern of elevation in CSE when TMS was delivered approximately 100 ms prior to median RT. In two of these experiments, we also found strong suppression of CSE when TMS was delivered at least 50 ms after median RT. The results show, for the first time, that a stimulus with a response history that conflicts with current task goals will elicit an initial elevation of CSE followed by CSE suppression shortly after, and the time of this change from excitation to suppression coincides with the time when the response would have been executed. Experiment 3 revealed that while the initial elevation in CSE occurs over a very specific timeframe, the subsequent suppression persists well after the point where participants have successfully withheld their response.

We interpret this pattern as a trade-off between automatic stimulus-driven excitation and the brain’s attempt to correct for this action tendency by suppressing the excitability of the motor system in line with current task goals. These results complement work on automatic priming of motor activity using EEG (e.g. Alamia et al. 2019; corticospinal excitability (McNair et al. 2017, Tran et al. 2020a) and behavior (e.g. Livesey and Costa 2014), in showing that cues can automatically elicit motor excitability that is concomitant with a preparedness to respond. Under the conditions of this study, participants were generally good at withholding their responses to the targets, as is evident from the relatively low proportion of commission errors in the presence of the target. Nevertheless, application of TMS allows us to examine underlying tendencies that contribute to these errors when they occur.

When we compared test trials with and without TMS, commission error rates were higher when TMS was delivered, and this effect was significantly larger during target presentations than control images. Further, this elevation in commission error rates to target images was greatest when TMS was delivered just prior to median RT (−100 ms in Experiments 1 and 3, −50 ms in Experiment 2). The timing-specific effect of TMS on commission errors to targets is most clearly seen in Fig. 4. These results suggest a super-additive interaction between the transient excitation elicited by the target stimulus and that produced by the TMS pulse. Since the use of TMS to measure CSE necessarily requires excitatory input to the motor system, it is possible that the conditioned action tendency elicited by the target image excites the motor system to a level that is close to the threshold required to produce an observable response and TMS delivery provides an external and sufficient source of motor excitation to pass this threshold.

**Fig. 6:**
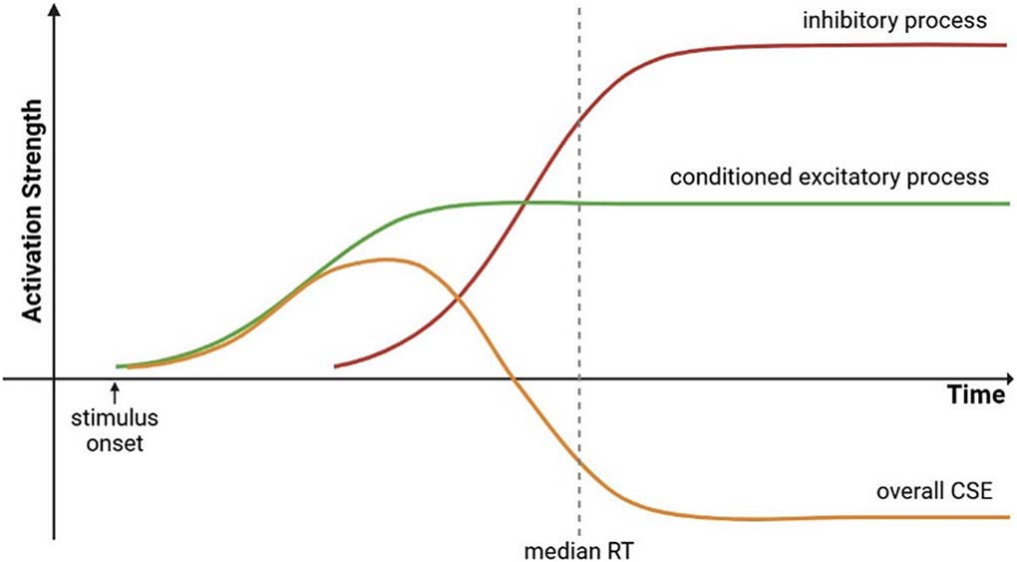
Conditioned excitatory and inhibitory processes contributing to overall corticospinal excitability.

If measured motor excitability is conceptualized as the summed output of independent underlying excitatory and inhibitory processes, our results are consistent with an early conditioned excitatory process that is engaged when participants see action cues combined with a slower inhibitory process engaged to counteract that conditioned excitation (for a similar concept in automatic stimulus processing, see Posner and Snyder 1975). At early timepoints, the conditioned excitatory process ramps up before the inhibitory process is active, resulting in overall elevated motor excitability. However, once participants engage the inhibitory process, evidence for excitation is terminated and is followed by a lasting suppression of CSE as the inhibitory process persists beyond the end of the conditioned excitation (see Fig. 6).

While our task and findings are novel, the results complement previous attempts to examine CSE dynamics when participants engage in tasks that contain conflict between competing responses (for complementary investigations using EEG, see Derosiere et al. 2018 & Szucs et al. 2009). Verleger et al. (2009) applied TMS to both hemispheres while participants conducted a flanker task (Eriksen and Eriksen 1974), with timing of TMS based on the individual’s predetermined time point of maximum activation in favor of the incorrect response. They found a temporally synchronous reduction in MEP for the incorrect hand and an increase in CSE for the correct hand, which they interpreted as indicating strong functional integration of conflicting response tendencies in the human motor system. On incongruent flanker task trials, Michelet et al. (2010) found initial excitability increase for the (incorrect) flanker arrow response, before coordinated suppression for the incorrect response and excitability increase for the correct target response, suggesting that representations of competing potential responses are active in the motor system prior to a response (see van Campen et al., 2014 for similar results with the Simon task). Klein et al. (2014, see also Duque et al. 2016) manipulated proportion congruence in a flanker task, and delivered TMS at critical timepoints during and before presentation of the target cue. They identified a key role for CSE suppression in managing anticipated conflict, but also noted that suppression was already strong at the anticipated onset of the target, especially in blocks containing mostly incongruent trials. This could indicate an important difference in the way participants prepare for standard discrete trial procedures (like the flanker task) and the continuous performance task that we used in this study.

Reward, and anticipation of reward, is also known to affect excitability in motor cortex, possibly by influencing motor output at a late stage, influencing both movement invigoration and motor learning (Derosiere et al. 2025). Although participants were not motivated by monetary rewards to perform well in this task, there were less tangible rewards (winning points) delivered in a performance-dependent manner, with responses at early time points being more strongly associated with winning points. It is possible that anticipation of reward at different time points may have some influence over motor excitability. Future research could thus examine whether the application of rewards in a time-dependent manner influences the strength of early CSE facilitation and late suppression.

While this study provides some initial insights into the temporal dynamics of action tendencies and their controlled suppression, questions remain about the nature of the inhibitory process that generates this suppression effect. Unlike other indices of cortical inhibition in motor cortex (e.g. see Loomes et al. 2023) we found no evidence that any aspect of this suppression was related to behavioral stopping efficiency as indexed using the stop signal task (see supplemental materials). The relatively slow time-course of this suppression suggests that it may be more related to a selective action cancellation process rather than early global action suppression (see Diesburg and Wessel 2021; Wadsley et al. 2022 for recent variants of this distinction). However, this issue could be addressed in future experiments that measure the somatotopic specificity of the suppression of CSE we have identified here. For instance, it may be that the early CSE increase is specific to the effector that has been used during training, whereas CSE suppression is more global and observed across other effectors. We believe that the suppression is triggered reactively, recruited to counteract an unwanted rise in excitation elicited by cues previously associated with action. This contrasts with a proactive inhibitory mechanism that should produce a suppression of CSE across all trials—during both target and control images—but such a process cannot be identified with the current experimental design. Moreover, the strength of a reactive inhibitory process could be dependent on the strength of the conditioned action tendency itself. Future work could investigate this by testing whether manipulations known to affect conditioned tendencies (e.g. extinction) have similar or dissociable effects on these facilitative and suppressive CSE effects.

## Supplementary Material

Chan_TimecourseActionTendencies_Supp_Materials_bhaf283
