# Supplementary material for: The time-course of action control: measuring conditioned action tendencies and action suppression using transcranial magnetic stimulation
Source: Cereb Cortex. 2025 Oct 15;35(10):bhaf283. doi: 10.1093/cercor/bhaf283 (PMC12527342; doi:10.1093/cercor/bhaf283)
Supplement: Chan_TimecourseActionTendencies_Supp_Materials_bhaf283 [file chan_timecourseactiontendencies_supp_materials_bhaf283.docx]

**Supplemental Materials**

**The time-course of action control: Measuring conditioned action tendencies and action suppression using transcranial magnetic stimulation**

Yvonne Y. Chan, Dominic M. D. Tran, Justin A. Harris, & Evan J. Livesey*

School of Psychology, The University of Sydney, Sydney, Australia, 2000

***Corresponding author:**

Evan J. Livesey

Address: School of Psychology,

Griffith Taylor Building A19,

The University of Sydney,

Sydney, Australia, 2006

Email: [evan.livesey@sydney.edu.au](mailto:evan.livesey@sydney.edu.au)

## Stop Signal Task

Participants in Experiments 1 and 2 completed two 96-trial blocks of a visual stop signal task. On each trial, a black fixation dot was presented for 500ms before the onset of a black arrow pointing left or right (4.2° × 3.2° of visual angle, 1500ms duration) acting as the Go signal. All stimuli were presented on a grey background. Participants were instructed to press the corresponding arrow key using the index and middle finger of their dominant hand as quickly and accurately as possible. On 25% of trials, a blue square (1.5° × 1.5° of visual angle) appeared overlaid on the arrow after a stop signal delay (SSD) and acted as a signal for the participant to withhold their response. The initial stop signal delay was 250 ms after the onset of the Go signal, and was adjusted using a 50% staircasing procedure: the SSD was increased by 50ms after a successful stop and decreased by 50ms after an unsuccessful stop to ensure an overall stopping accuracy rate of approximately 50%.

Overall SSRT was calculated using the integration method (Verbruggen et al., 2019) and averaged across blocks. Blocks with a stopping accuracy rate > 75% or < 25%, blocks which violated the independent race model (Verbruggen et al., 2019), and blocks in which participants demonstrated excessive slowing (>2 ms per trial) were excluded from analysis.

### Results

Three additional participants in both Experiment 1 and Experiment 2 were excluded from analysis for not having at least one valid SST block after block exclusions were applied.

**Figure S1.** Correlation between SSRT and log-normalised MEPs at the four timepoints in Experiments 1 and 2.


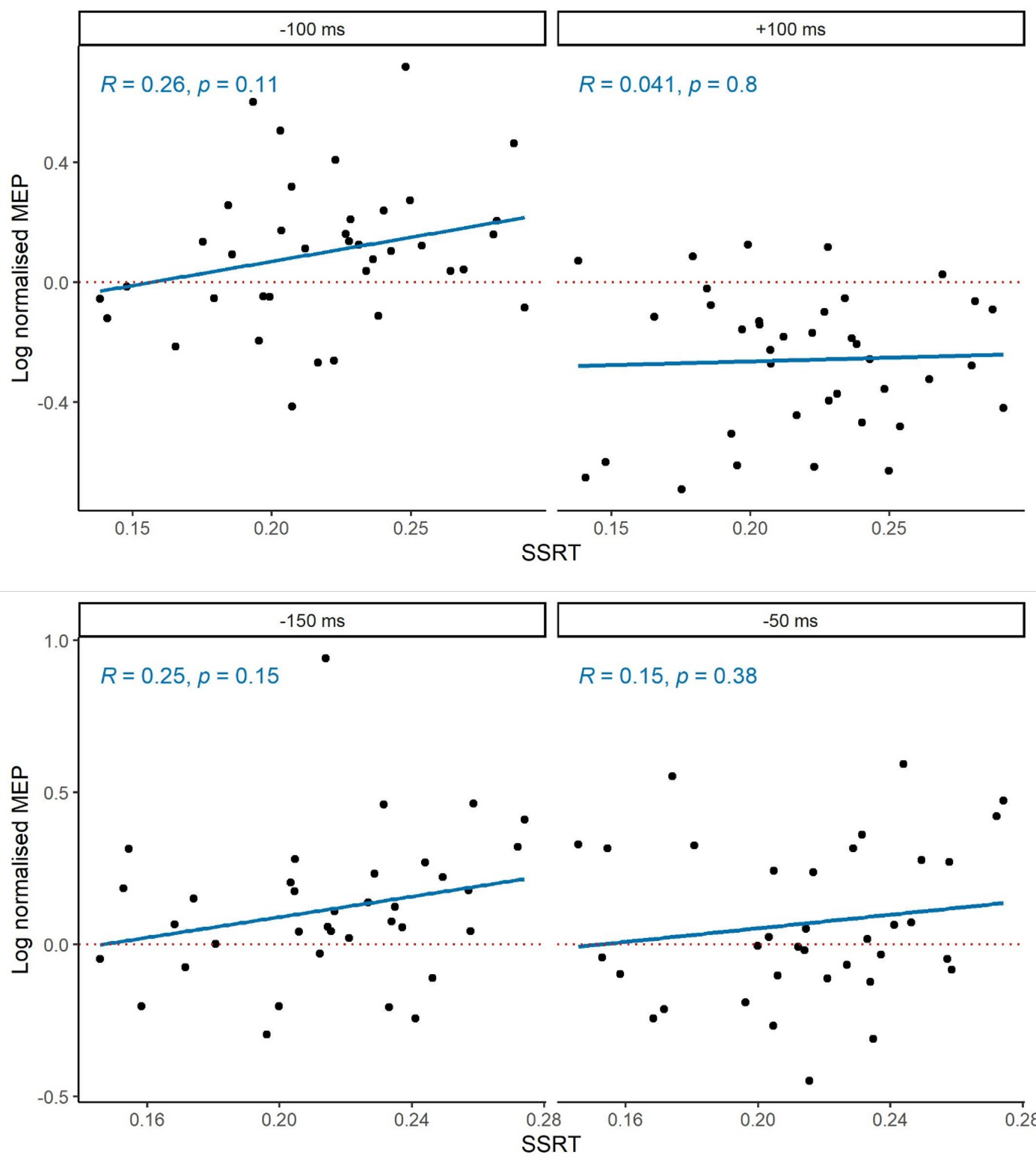


## Experiment 3 t-tests

Table S1 reports the outcome of a series of one-sample t-tests comparing log normalised MEP scores against a value of 0, for each time point tested in Experiment 3. A score of zero reflects equivalent MEP amplitude for control and target stimuli, whereas scores above and below zero suggest enhanced and suppressed MEPs respectively, for the target stimulus relative to the control.

| **Table S1.** | | | | |
| --- | --- | --- | --- | --- |
| **TMS time** | **t** | **df** | **p** | **d** |
| -0.2 | 0.677 | 31 | 0.503 | .120 |
| -0.15 | 2.816 | 31 | 0.008 | .498 |
| -0.1 | 4.594 | 31 | < .001 | .812 |
| -0.05 | 0.508 | 31 | 0.615 | .090 |
| 0 | -2.121 | 31 | 0.042 | -.375 |
| 0.05 | -5.079 | 31 | < .001 | -.898 |
| 0.1 | -4.310 | 31 | < .001 | -.762 |
| 0.2 | -3.125 | 31 | 0.004 | -.552 |
| 0.3 | -2.205 | 31 | 0.035 | -.390 |
| 0.4 | -4.739 | 31 | < .001 | -.838 |
|  | | | | |
| *Note.* For the Student t-test, the alternative hypothesis specifies that the mean is different from 0. To correct for multiple comparisons, test are only considered significant if p < .005. | | | | |

**Analysis of pre-TMS EMG activity**

To test for variations in pre-TMS motor activity in the EMG record, we calculated root mean squared difference (RMSD) over the interval spanning 100 ms to 5 ms prior to delivery of the TMS pulse. We then analysed this RMSD variable by running repeated measures ANOVA in each experiment, with stimulus type and TMS time as within-subjects factors.

In Experiment 1, there was no main effect of TMS time, *F*(1,40) = 0.31, *p* = 0.583, *η_p_^2^* = 0.0009, no main effect of stimulus type, *F*(1,40) = 1.70 *p* = 0.199, *η_p_^2^* = 0.0031, and no interaction, *F*(1,40) = 1.62, *p* = 0.210, *η_p_^2^* = 0.0045, indicating that RMSD in the pre-TMS EMG record did not significantly vary by condition.

In Experiment 2, there was no main effect of TMS time, *F*(1,38) = 1.06, *p* = 0.309, *η_p_^2^* = 0.0009, but this time we did observe a main effect of stimulus type, *F*(1,38) = 8.98, *p* = 0.005, *η_p_^2^* = 0.0087, qualified by an interaction between TMS time and stimulus type, *F*(1,38) = 4.90, *p* = 0.033, *η_p_^2^* = 0.0045. Follow up analyses examining the effect of stimulus type at each TMS timepoint indicated that the pre-TMS RMSD did not differ between target and control trials when TMS was delivered 150 ms before median RT, *t*(38) = 0.74, *p* = 0.46. However, on trials where TMS was delivered 50 ms before median RT, RMSD was significantly great for the target trials than the control trials, *t*(38) = 3.24, *p* = 0.002.

In Experiment 3, the same analysis was run but note that TMS time now included 10 levels spanning 200 ms before to 400 ms after median RT. Greenhouse Geisser corrections were used to adjust for violations of sphericity. There was no main effect of TMS time, *F*(4.16,129) = 1.836, *p* = .123, *η_p_^2^* = 0.056, no main effect of stimulus type, *F*(1,31) = 1.81 *p* = 0.189, *η_p_^2^* = 0.055, and no significant interaction between these factors, *F*(4.3,133) = 1.93, *p* = 0.104, *η_p_^2^* = 0.059.

It seems that over the three experiments, only one experiment, and indeed only one timepoint (50 ms prior to median RT, Experiment 2) showed significant differences in pre-TMS RMSD. Although this result is in the direction consistent with heightened pre-TMS muscle activity in the presence of the target stimulus, we note that the strongest evidence for CSE elevation in Experiment 2 actually came from the earlier time point (stimulation 150 ms prior to median RT), where there was no evidence of elevated pre-TMS RMSD. On the whole, we do not think there is strong evidence that pre-TMS activity can account for the observed changes in MEP amplitudes across the experiments.

**Effect of Response Errors on stimulus-driven MEP effects**

Our primary analyses of MEPs in the test phase included trials in which the participant ultimately made a response in error to a target or control stimulus. Although these response error trials were relatively infrequent, comprising fewer than 5% of trials overall, their frequency varied considerably over stimulus type and TMS time conditions, and were more prevalent on target stimulus trials with TMS delivered at early timepoints prior to median RT. This raises the possibility that MEPs were elevated at these early timepoints in the target condition because participants were about to respond when TMS was delivered. If participants were already responding at the time of TMS delivery then their deliberate muscle movements could contribute to the amplitude of the MEP. However, this remains highly unlikely because trials with large noise in the EMG record prior to the TMS pulse were removed from analysis (indeed the pre-TMS activity analysis reported above confirms this). On the other hand, if participants had not yet initiated a muscle response but were about to at the time of TMS, this may result in CSE elevation (e.g. see Poole et al., 2018). Although this is a possibility, removing trials with responses from analysis could also produce biases in the results that may lead to underestimating a CSE elevation effect. It is clear from the results of Experiments 1 and 2 that the TMS delivered early (around 100 ms prior to median RT) *causes* responses when the target stimulus is presented, that is, in the presence of the conditions that we expect there to be a conditioned action tendency in play. This means removing MEPs from trials with responses is likely to remove the trials with the strongest action tendency.

To address this, we tried two analyses focused on the early time points that reveal the strongest evidence of MEP elevation (that is, trials with TMS delivered at the -100 and -150 ms timepoints). To maximise power, we collapsed across these two timepoints and across experiment for this analysis, providing a total N=112. First, we sought to examine whether the presence of a response error and the type of stimulus (target vs control) predicted MEP amplitude. This analysis is complicated by the very low response rate on control trials relative to target trials. Analysis of log normalised MEPs was deemed unsuitable for this analysis as only 15 participants made even a single error to control stimuli at the early TMS time points in question. Instead, we took the log of each individual MEP amplitude and ran a linear mixed model analysis with participant as random factor and stimulus type (target vs control) and response error (present vs absent) as fixed factors. The model yielded significant effects of stimulus type, β = 0.376, SE = 0.169, *df* = 5748.20, *t* = 2.227, *p* = 0.0260, indicating larger MEPs in the presence of target stimuli relative to control, and a significant effect of response error, β = 0.360, SE = 0.162, *df* = 5748.97, *t* = 2.216, *p* = 0.0267. The interaction did not reach significance, β = 0.308, SE = 0.170, *df* = 5748.43, *t* = 1.811, *p* = 0.070. However, the substantial correlation between stimulus type and response error means this result should be interpreted with caution.

Having established that stimulus type appears to explain variance in MEP amplitude when response errors are present in the model, we then removed trials containing a response error and re-ran an analysis of the log normalised MEPs. If the log normalised values are greater than 0 then it suggests evidence of elevated CSE in the absence of response errors. This analysis yielded *t*(111) = 3.599, *p* < 0.001, *d* = .340, indicating overall strong evidence of CSE facilitation. Note that, as discussed, we believe this analysis substantially *underestimates* the effect of stimulus type on corticospinal excitability because TMS appears to trigger responses that would not otherwise be made when there is a stimulus-elicited action tendency. When we repeat the analysis with response trials included, it yields *t*(111) = 5.623, *p* < 0.001, *d* = .531

If, in the presence of early TMS, it is the underlying CSE changes that cause elevation of MEPs in the presence of the target and, as a downstream consequence, also causes the triggering of responses, then one would expect those individuals who respond more to the target at this time point to also show greater CSE elevation even when they do not make a response. To test this we ran a Pearson bivariate correlation on the log normalised MEP index calculated on trials without a response (described above) with response rate in the presence of the target stimuli, calculated on the same TMS timepoints. This yielded a correlation of *r* = 0.19, *p* = .047, *df* = 110. When we include response trials, the correlation between log normalised MEPs and response rate increases to *r* = 0.38, *p* < .001, *df* = 110.

**Mean number of trials per condition.**

Table S2 reports the mean number of trials with valid MEPs (i.e. those used in analyses), mean number of invalid MEP trials (rejected for having excessive pre-TMS EMG noise or for other technical reasons such as the TMS machine failing to trigger), and mean number of MEPs excluded from analysis as their amplitude was less than 50 μV.

**Table S2.**

| Experiment | Stimulus Type | TMS time | N include (valid MEPs) | N exclude invalid | N exclude  < 50μV |
| --- | --- | --- | --- | --- | --- |
| 1 | Target | -100 ms | 28.5 (2.96) | 2.9 (2.67) | 0.56 (1.91) |
|  |  | +100 ms | 27.6 (3.38) | 3.54 (2.91) | 0.83 (1.48) |
|  | Control | -100 ms | 29.7 (2.73) | 2.12 (2.71) | 0.22 (0.57) |
|  |  | +100 ms | 29.8 (2.88) | 2.02 (2.75) | 0.17 (0.38) |
| 2 | Target | -150 ms | 28.8 (4.05) | 2.79 (3.68) | 0.44 (0.99) |
|  |  | -50 ms | 26.3 (3.85) | 5.21 (3.65) | 0.51 (0.82) |
|  | Control | -150 ms | 29.1 (3.36) | 2.33 (2.7) | 0.59 (1.19) |
|  |  | -50 ms | 29.3 (3.11) | 2.18 (2.66) | 0.51 (1.07) |
| 3 | Target | -200 ms | 9.44 (0.76) | 0.47 (0.72) | 0.09 (0.3) |
|  |  | -150 ms | 9.72 (0.58) | 0.28 (0.58) | 0.00 (0.00) |
|  |  | -100 ms | 9.00 (1.05) | 0.94 (1.05) | 0.06 (0.25) |
|  |  | -50 ms | 8.06 (1.39) | 1.78 (1.43) | 0.16 (0.45) |
|  |  | 0 ms | 7.97 (1.43) | 1.81 (1.33) | 0.22 (0.49) |
|  |  | 50 ms | 8.78 (1.29) | 1.00 (1.08) | 0.22 (0.55) |
|  |  | 100 ms | 8.97 (1.31) | 0.72 (0.99) | 0.31 (0.64) |
|  |  | 200 ms | 8.84 (1.22) | 0.94 (1.13) | 0.22 (0.49) |
|  |  | 300 ms | 9.09 (1.17) | 0.81 (1.09) | 0.09 (0.30) |
|  |  | 400 ms | 9.34 (0.79) | 0.56 (0.76) | 0.09 (0.30) |
|  | Control | -200 ms | 9.69 (0.74) | 0.28 (0.63) | 0.03 (0.18) |
|  |  | -150 ms | 9.78 (0.49) | 0.22 (0.49) | 0.00 (0.00) |
|  |  | -100 ms | 9.34 (0.83) | 0.47 (0.72) | 0.19 (0.47) |
|  |  | -50 ms | 9.72 (0.77) | 0.25 (0.76) | 0.03 (0.18) |
|  |  | 0 ms | 9.41 (1.01) | 0.41 (0.84) | 0.19 (0.47) |
|  |  | 50 ms | 9.59 (0.67) | 0.34 (0.65) | 0.06 (0.25) |
|  |  | 100 ms | 9.25 (0.92) | 0.66 (0.87) | 0.09 (0.39) |
|  |  | 200 ms | 9.56 (0.67) | 0.41 (0.61) | 0.03 (0.18) |
|  |  | 300 ms | 9.56 (0.88) | 0.38 (0.83) | 0.06 (0.25) |
|  |  | 400 ms | 9.66 (0.60) | 0.28 (0.52) | 0.06 (0.25) |
